# Fibroblast growth factor 23 dysregulates late sodium current and calcium homeostasis with enhanced arrhythmogenesis in pulmonary vein cardiomyocytes

**DOI:** 10.18632/oncotarget.12470

**Published:** 2016-10-04

**Authors:** Shih-Yu Huang, Yao-Chang Chen, Yu-Hsun Kao, Ming-Hsiung Hsieh, Yung-Kuo Lin, Cheng-Chih Chung, Ting-I Lee, Wen-Chin Tsai, Shih-Ann Chen, Yi-Jen Chen

**Affiliations:** ^1^ Graduate Institute of Clinical Medicine, College of Medicine, Taipei Medical University, Taipei, Taiwan; ^2^ Division of Cardiology, Department of Internal Medicine, Cathay General Hospital, Taipei, Taiwan; ^3^ Department of Biomedical Engineering, National Defense Medical Center, Taipei, Taiwan; ^4^ Department of Medical Education and Research, Wan Fang Hospital, Taipei Medical University, Taipei, Taiwan; ^5^ Division of Cardiovascular Medicine, Department of Internal Medicine, Wan Fang Hospital, Taipei Medical University, Taipei, Taiwan; ^6^ Division of Cardiology, Department of Internal Medicine, School of Medicine, College of Medicine, Taipei Medical University, Taipei, Taiwan; ^7^ Department of General Medicine, School of Medicine, College of Medicine, Taipei Medical University, Taipei, Taiwan; ^8^ Division of Endocrinology and Metabolism, Department of Internal Medicine, Wan Fang Hospital, Taipei Medical University, Taipei, Taiwan; ^9^ Division of Cardiology, Tzu-Chi General Hospital, Institute of Medical Sciences, Tzu-Chi University, Hualien, Taiwan; ^10^ Division of Cardiology and Cardiovascular Research Center, Veterans General Hospital-Taipei, Taipei, Taiwan

**Keywords:** atrial fibrillation, calcium regulation, fibroblast growth factor-23, pulmonary vein, late sodium current, Pathology Section

## Abstract

Fibroblast growth factor 23 (FGF23), elevated in chronic renal failure, increases atrial arrhythmogenesis and dysregulates calcium homeostasis. Late sodium currents (I_Na-Late_) critically induces ectopic activity of pulmoanry vein (the most important atrial fibrillation trigger). This study was to investigate whether FGF23 activates the I_Na-Late_ leading to calcium dysregulation and increases PV arrhythmogenesis. Patch clamp, western blot, and confocal microscopy were used to evaluate the electrical activities, calcium homeostasis, and mitochondrial reactive oxygen species (ROS) in PV cardiomyocytes with or without FGF23 (0.1 or 1 ng/mL) incubation for 4~6 h. Compared to the control, FGF23 (1 ng/mL, but not 0.1 ng/mL)-treated PV cardiomyocytes had a faster beating rate. FGF23 (1 ng/mL)-treated PV cardiomyocytes had larger I_Na-Late_, calcium transients, and mitochondrial ROS than controls. However, ranolazine (an inhibitor of I_Na-Late_) attenuated FGF23 (1 ng/mL)-increased beating rates, calcium transients and mitochondrial ROS. FGF23 (1 ng/mL)-treated PV cardiomyocytes exhibited larger phosphorylation of calcium/calmodulin-dependent protein kinase II (CaMKII). Chelerythrine chloride (an inhibitor of protein kinase C) decreased I_Na-Late_ in FGF23 (1 ng/mL)-treated PV cardiomyocytes. However, KN93 (a selective CaMKII blocker) decreased I_Na-Late_ in control and FGF23 (1 ng/mL)-treated PV cardiomyocytes to a similar extent. In conclusion, FGF23 increased PV arrhythmogenesis through sodium and calcium dysregulation by acting protein kinase C signaling.

## INTRODUCTION

Atrial fibrillation (AF) is the most often seen cardiac arrhythmia to cause heart failure and cardiovascular events in clinical practice. [1, 2] A previous study reported that fibroblast growth factor 23 (FGF23), a regulatory hormone secreted by osteocytes for phosphate-calcium homeostasis, can increase the incidence of AF. [3–5] High circulating levels of FGF23 in chronic kidney disease (CKD) linked to an elevated prevalence of AF. [6–9] FGF23 was shown to induce atrial arrhythmogenesis due to calcium dysregulation with increased phosphorylation of calcium/calmodulin-dependent protein kinase II (CaMKII). [10–13] Pulmonary vein (PV) is the most important focus of initiating AF. [14–16] Our previous animal study showed that CKD PVs had increased arrhythmogenesis. [17] Accordingly, FGF23 is hypothesized to induce the high incidence of AF in CKD patients through increasing PV electrical activity.

FGF23 is known to produce cardiac hypertrophy by activating calcineurin with concomitant promotion of protein kinase C (PKC), which can increase late sodium current (I_Na-Late_) in the cardiomyocyte. [11, 18–22] Moreover, FGF23 increases cellular reactive oxygen species (ROS). [23] Mitochondria ROS are the mian source of cellular ROS in the cardiomyocyte. [24] Up-regulation of I_Na-Late_ can lead to overproduction of mitochondrial ROS and oxidation of CaMKII, resulting in abnormalities of calcium handling. [25] The purpose of this study was to investigate whether FGF23 increases PV arrhythmogenesis and the potential underlying mechanisms.

## RESULTS

**Figure 1:**
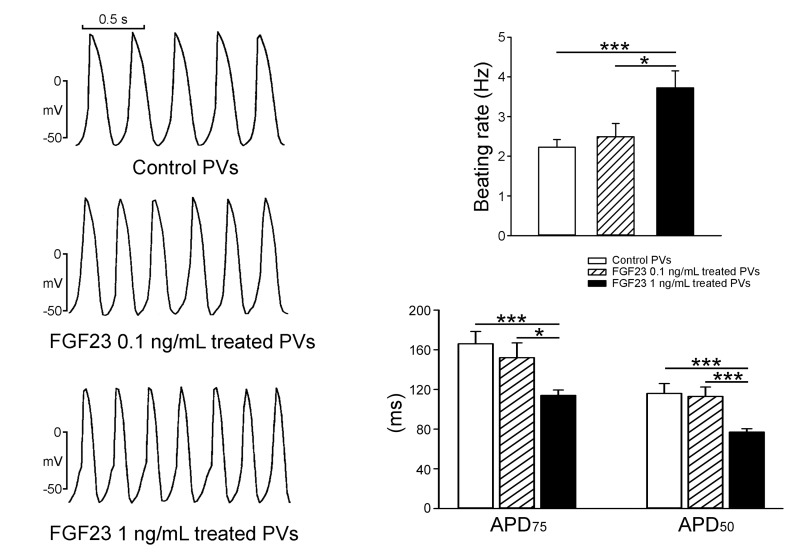
Spontaneous beating rates and action potential duration (APD) of pulmonary vein (PV) cardiomyocytes in the control and different concentrations of FGF23 An example and average data show that FGF23 (1 ng/mL)-treated PV cardiomyocytes (*n* = 10) had more-rapid beating rates and shorter APD than control (*n* = 10) or FGF23 (0.1 ng/mL)-treated PV cardiomyocytes (*n* = 10). * *p* < 0.05, *** *p* < 0.005 *vs*. the control.

**Figure 2:**
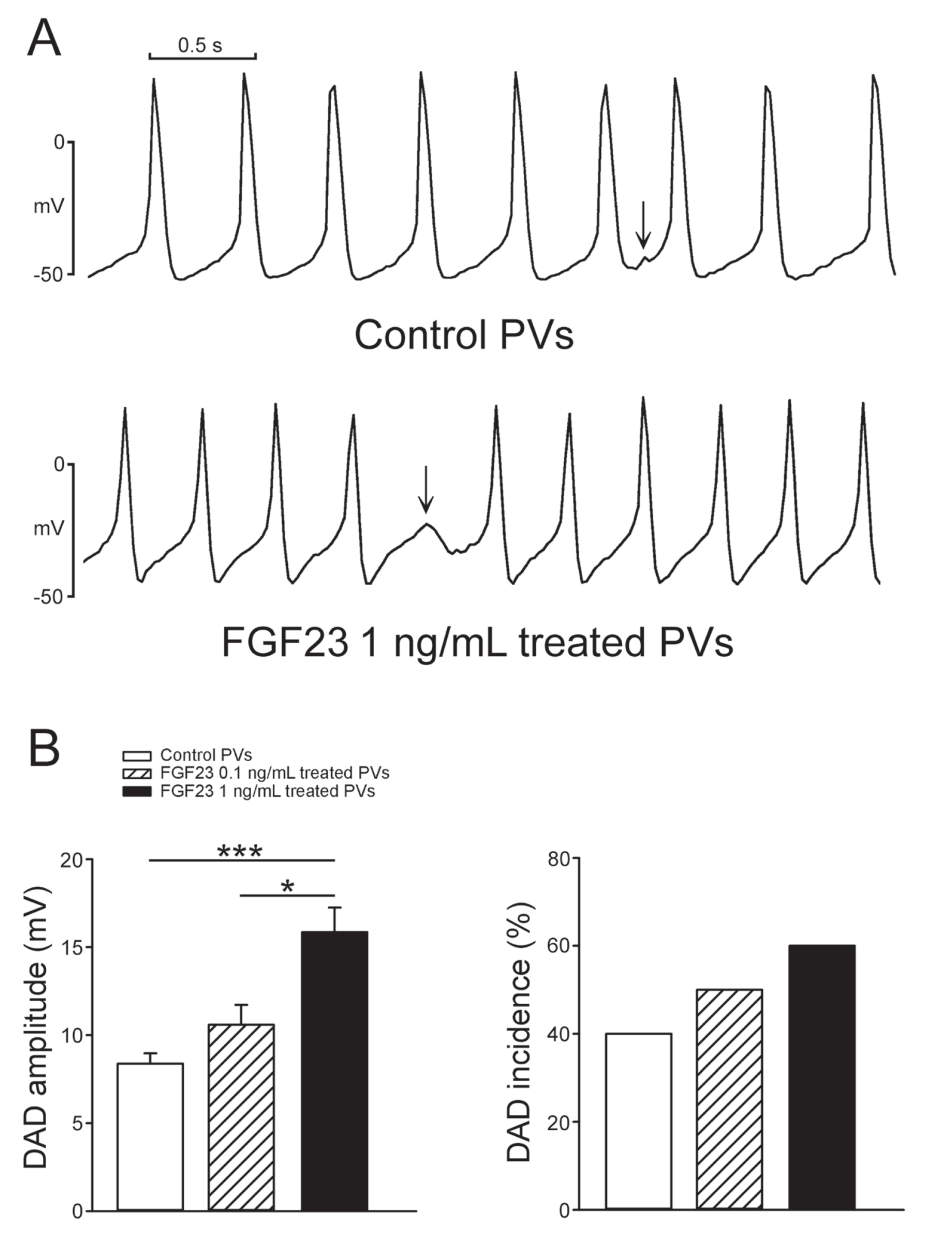
The incidence and amplitude of delayed afterdepolarizations (DADs) in control and FGF23-treated PV cardiomyocytes **A.** Tracings show that a FGF23 (1 ng/mL)-treated PV cardiomyocyte had a larger amplitude of DAD (arrow) than a control PV cardiomyocyte. **B.** In the cells with DADs, and FGF23 (1 ng/mL, *n* = 6)-treated PV cardiomyocytes had larger amplitude of DAD than control (*n* = 4) and FGF23 (0.1 ng/mL, *n* = 5)-treated PV cardiomyocytes. However, the incidence of DAD was not significantly different in control (*n* = 10), and FGF23 (0.1 ng/mL, *n* = 10) and FGF23 (1 ng/mL, *n* = 10)-treated PV cardiomyocytes.* *p* < 0.05, *** *p* < 0.005.

**Figure 3:**
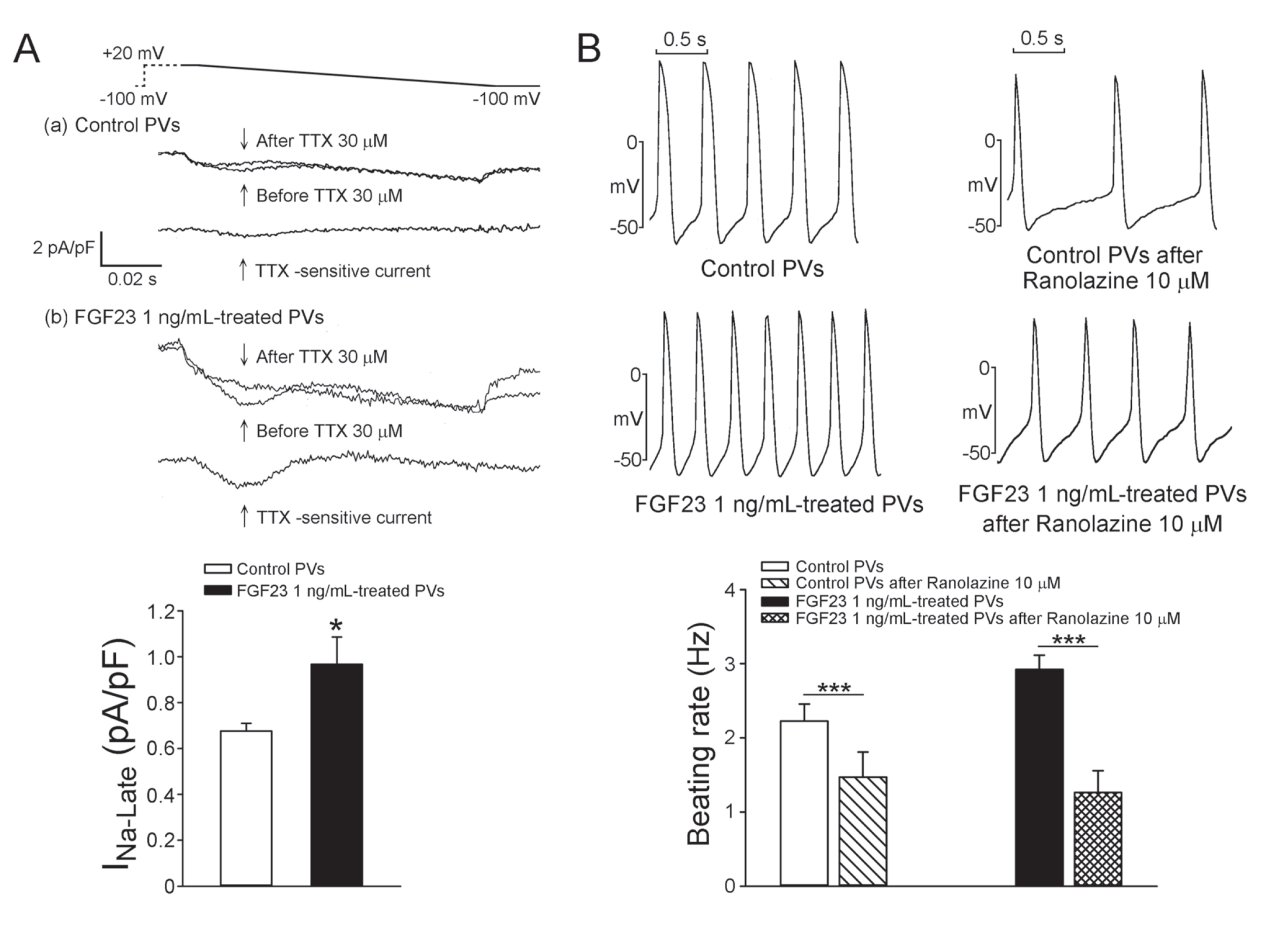
The *I*_**Na-Late**_ in control and FGF23-treated PV cardiomyocytes and the effect of ranolazine on the spontaneous beating rates of control and FGF23-treated PV cardiomyocytes **A.** Tracings and average data of the *I*_Na-Late_ from control (*n* = 18) and FGF23 (1 ng/mL)-treated PV cardiomyocytes (*n* = 18). **B.** An example and average data show that control (*n* = 8) and FGF23 (1 ng/mL)-treated PV cardiomyocytes (*n* = 8) after ranolazine (10 μM) had slower beating rates than those before ranolazine. * *p* < 0.05, *** *p* < 0.005.

As shown in Figure 1, FGF23 (1 ng/mL)-treated PV cardiomyocytes had faster beating rate than control or FGF23 (0.1 ng/mL)-treated PV cardiomyocytes. FGF23 (1 ng/mL)-treated PV cardiomyocytes had shorter action potential duration (APD) at 75% and 50% repolarization of the amplitude (APD_75_ and APD_50_) than control and FGF23 (0.1 ng/mL)-treated PV cardiomyocytes. FGF23 (1 ng/mL)-treated PV cardiomyocytes had higher amplitude of delayed afterdepolarizations (DADs) than control or FGF23 (0.1 ng/mL)-treated PV cardiomyocytes (Figure 2). However, control and FGF23 (0.1 and 1 ng/mL)-treated PV cardiomyocytes had similar incidence of DAD. Moreover, FGF23 (1 ng/mL)-treated PV cardiomyocytes had a larger I_Na-Late_ than control PV cardiomyocytes (Figure 3A). Ranolazine (10 μM, an inhibitor of the I_Na-Late_) reduced the beating rate in control and FGF23 (1 ng/mL)-treated PV cardiomyocytes (Figure 3B). The ranolazine-reduced beating rate was larger in FGF23 (1 ng/mL)-treated PV cardiomyocytes than in control PV cardiomyocytes.

**Figure 4:**
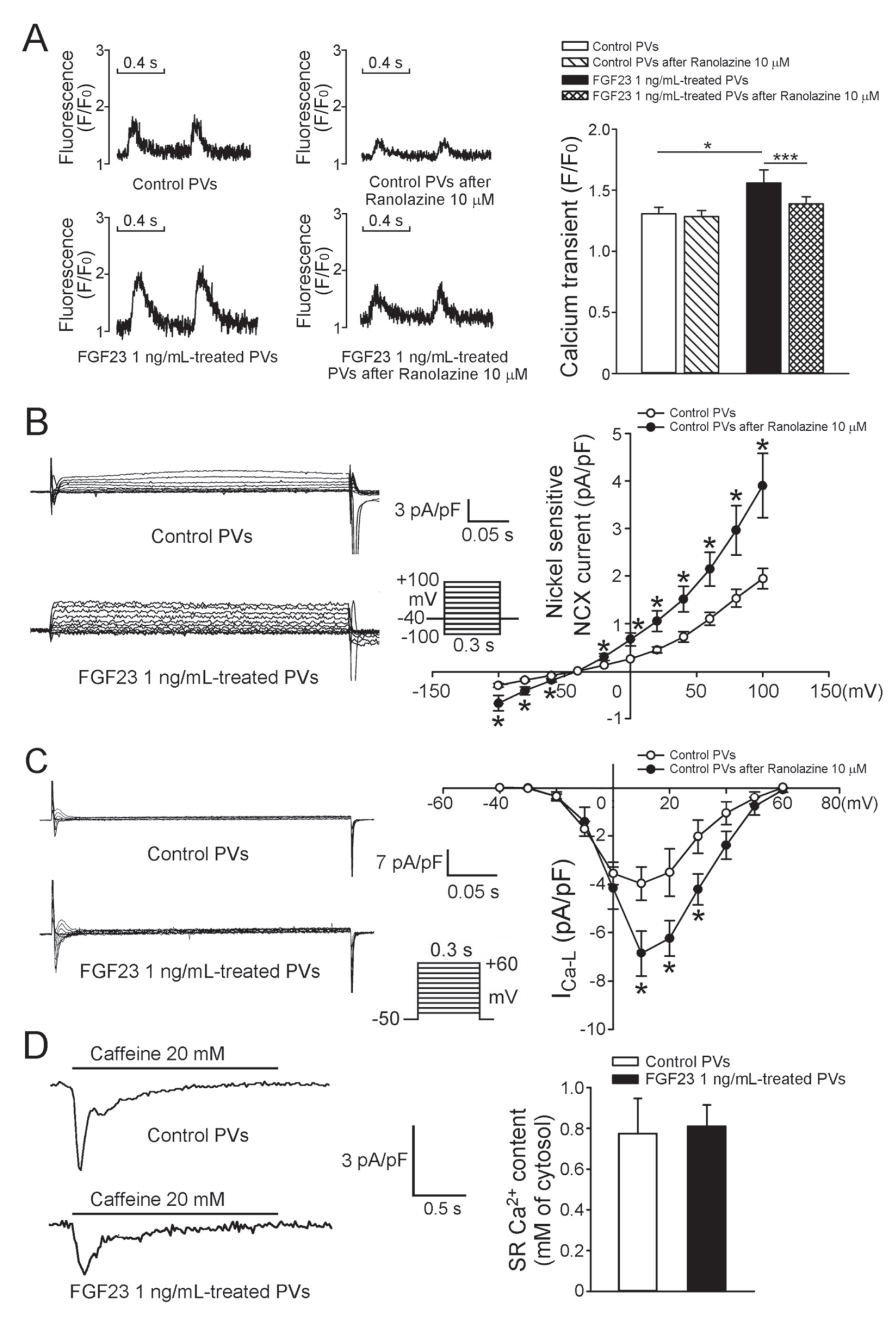
Intracellular calcium homeostasis and ionic currents in control and FGF23-treated PV cardiomyocytes **A.** Tracings and average data of calcium transients in control (*n* = 10) and FGF23 (1 ng/mL)-treated PV cardiomyocytes (*n* = 10) before and after ranolazine (10 μM) administration. **B.** Tracings and I-V relationship of the NCX from control (*n* = 10) and FGF23 (1 ng/mL)-treated PV cardiomyocytes (*n* = 10). **C.** Tracings and I-V relationship of the I_Ca-L_ from control (*n* = 10) and FGF23 (1 ng/mL)-treated PV cardiomyocytes (*n* = 10). The inset in the current trace shows the clamp protocol. **D.** Tracings and average data of caffeine-induced NCX and SR calcium contents from integrating NCX currents in control (*n* = 11) and FGF23 (1 ng/mL)-treated PV cardiomyocytes (*n* = 11). * *p* < 0.05, *** *p* < 0.005.

We evaluated the effects of FGF23 (1 ng/mL) on calcium homeostasis and found that FGF23 (1 ng/mL)-treated PV cardiomyocytes had larger calcium transients than control PV cardiomyocytes (Figure 4A). Ranolazine (10 μM) reduced calcium transients in FGF23 (1 ng/mL)-treated PV cardiomyocytes, but not in control PV cardiomyocytes. In the presence of ranolazine, control and FGF23 (1 ng/mL)-treated PV cardiomyocytes had similar calcium transients. FGF23 (1 ng/mL)-treated PV cardiomyocytes had larger sodium-calcium exchangers (NCXs), and L-type calcium currents (I_Ca-L_) than control PV cardiomyocytes (Figure 4B, 4C). However, control and FGF23-treated PV cardiomyocytes had similar sarcoplasmic reticulum (SR) calcium contents (Figure 4D).

**Figure 5:**
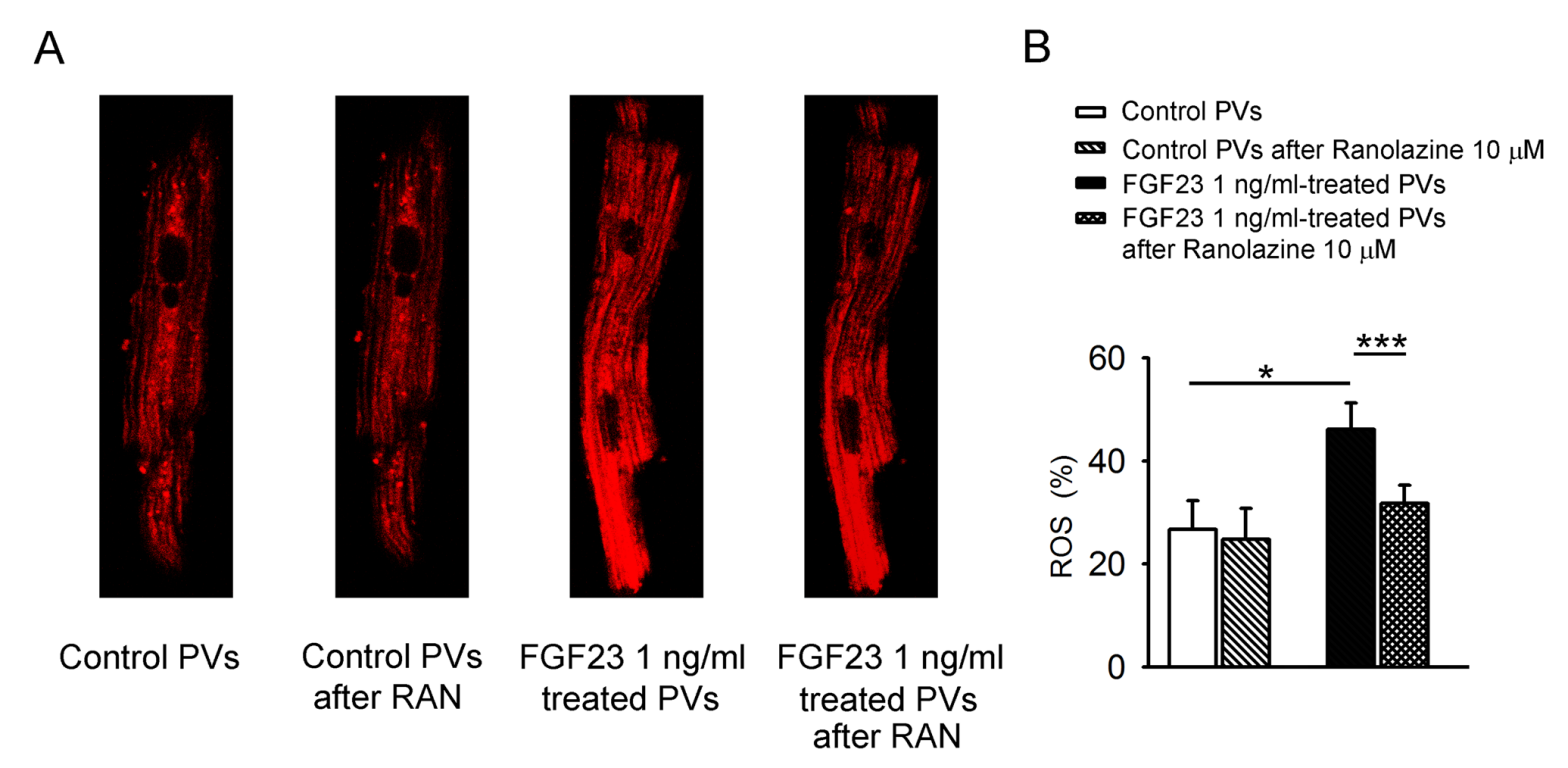
ROS in mitochondria of control and FGF23-treated PV cardiomyocytes **A.** An example and **B.** average data of mitochondrial levels of ROS in control (*n* = 8) and FGF23 (1 ng/mL)-treated PV cardiomyocytes (*n* = 8) before and after ranolazine (10 μM, RAN) administration. * *p* < 0.05.

Figure 5 shows the effect of FGF23 (1 ng/mL) on mitochondrial ROS, where FGF23 (1 ng/mL)-treated PV cardiomyocytes had higher mitochondrial ROS than control PV cardiomyocytes. Ranolazine (10 μM) decreased mitochondrial ROS in FGF23 (1 ng/mL)-treated PV cardiomyocytes, but not in control cardiomyocytes. Additionally, in the presence of ranolazine, control and FGF23 (1 ng/mL)-treated PV cardiomyocytes had similar mitochondrial ROS levels.

**Figure 6:**
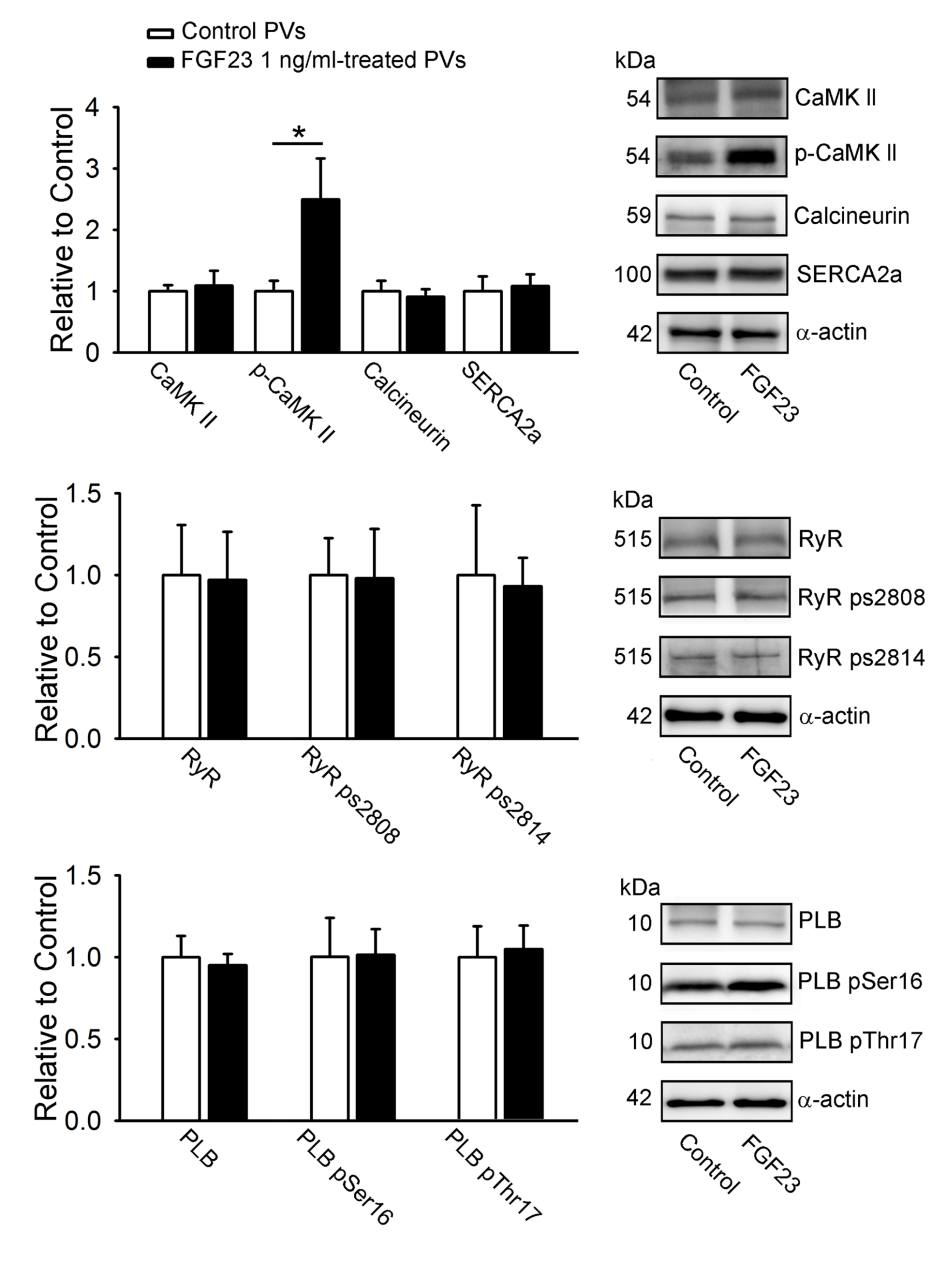
Calcium-handling proteins in FGF23-treated PV cardiomyocytes Representative immunoblot and average data of CaMKII, pCaMKII, calcineurin, SERCA2a, RyR channels, RyR ps2808, RyR ps2814, PLB, PLB pSer16, PLB pThr17 from control (*n* = 10) and FGF23 (1 ng/mL)-treated PV cardiomyocytes (*n* = 10). * *p* < 0.05 *vs*. the control.

As shown in Figure 6, FGF23 (1 ng/mL)-treated PV cardiomyocytes had larger phosphorylation of CaMKII (pCaMKII) compared to control PV cardiomyocytes. However, FGF23 (1 ng/mL)-treated and control PV cardiomyocytes had similar expressions of CaMKII, calcineurin, SR Ca^2+^ - ATPase (SERCA2a), ryanodine receptor channel (RyR), phospholamban (PLB), phosphorylated RyR at Ser-2808 and Ser-2814 (RyR pS2808, RyR pS2814), and phosphorylated PLB at Ser16 and Thr17 (PLB pSer16 and PLB pThr17).

**Figure 7:**
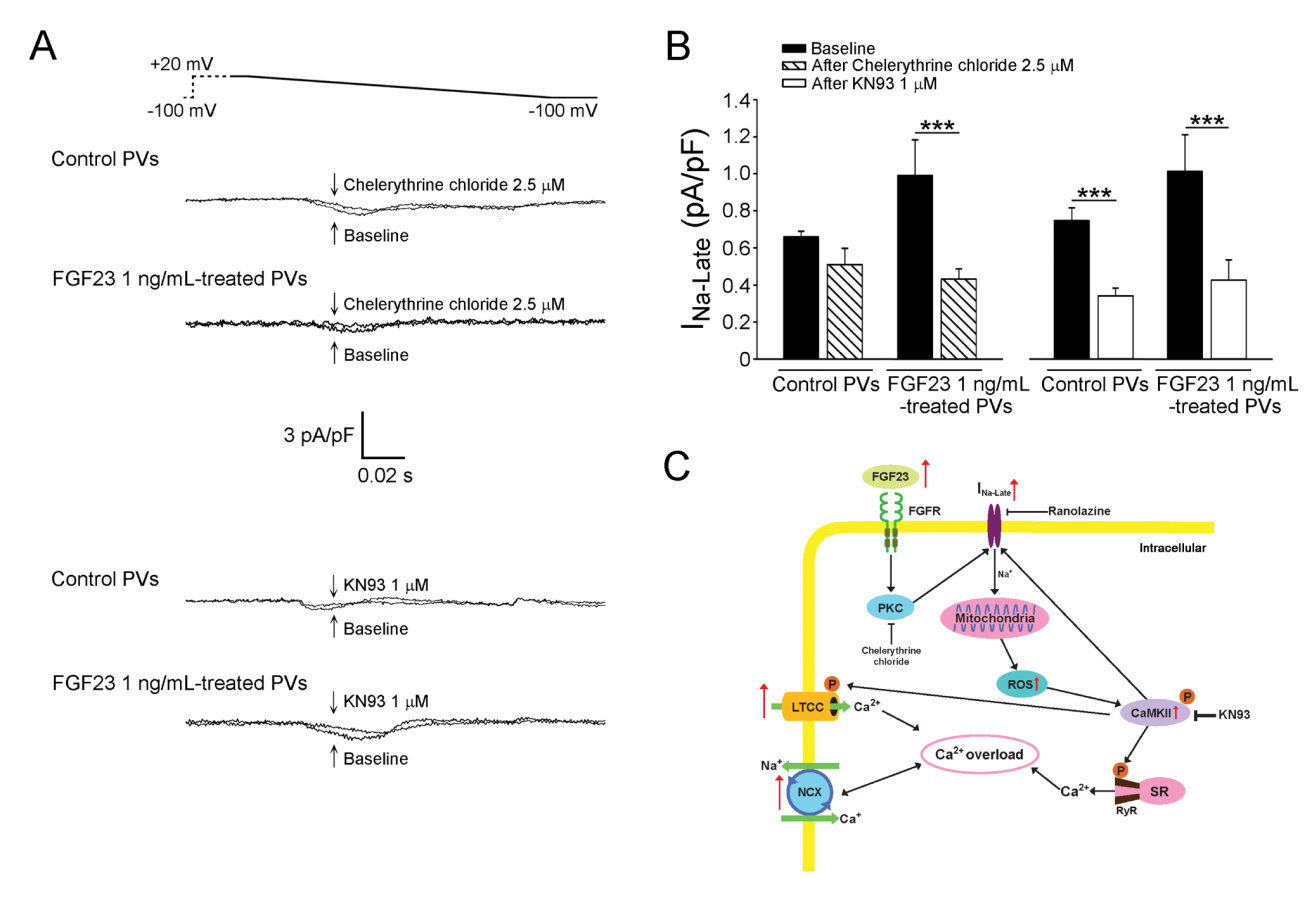
Signaling pathway and proposed mechanism of FGF23 on the modulation of *I*_**Na-Late**_ leading to calcium handling abnormalities **A.** Tracings and **B.** average data of the *I*_Na-Late_ from control (*n* = 8) and FGF23 (1 ng/mL)-treated PV cardiomyocytes (*n* = 8) before and after chelerythrine chloride (2.5 μM) or KN93 (1 μM) administration. *** *p* < 0.005. **C.** The *I*_Na-Late_ was activated by enhanced FGF23 through the signaling of PKC or the phosphorylation of CaMKII, which can be stimulated by increased mitochondrial ROS. FGF23-related PV arrhythmogenesis can be induced by calcium overload, which is produced by increased L-type calcium current (LTCC), NCX, and phosphorylation of CaMKII resulting in RyR dysfunction in the SR.

We studied the potential signaling underlying the FGF23-induced I_Na-Late_, whereas chelerythrine chloride (2.5 μM, an inhibitor of PKC) decreased the I_Na-Late_ in FGF23 (1 ng/mL)-treated PV cardiomyocytes, but not in control cardiomyocytes (Figure 7). However, KN93 (1 μM, a selective inhibitor of CaMKII) but not KN92 (1 μM, an inactive analog of KN93) decreased the I_Na-Late_ in control and FGF23 (1 ng/mL)-treated PV cardiomyocytes to a similar extent (−52.87%±3.33% vs. −57.42%±5.54%, *p* > 0.05).

## DISCUSSION

FGF23 was shown to enhance arrhythmogenesis in mice atrial cell lines (HL-1 cells) with increased triggered activity of DADs and calcium transient. [10] In the present study, we found that FGF23 at a concentration of 1 ng/mL increased PV spontaneous activity and amplitude of DADs, which may have been caused by FGF23-increased NCX currents. [26, 27] In addition, the shorter APD in FGF23 (1 ng/mL)-treated PV cardiomyocytes may be caused by their larger NCX currents (reverse mode) and faster beating rates (rate-dependent shortening). [28] Our results imply that FGF23 at 1 ng/mL may enhance atrial arrhythmic burden through its arrhythmogenic potential in PV cardiomyocytes.

The increase of I_Na-Late_ can induce calcium overload through calcium entry from the NCX in PV cardiomyocytes and lead to arrhythmogenesis. [29–31] We found that FGF23 increased the I_Na-Late_ in PV cardiomyocytes, and ranolazine inhibited the effects of FGF23 on PV arrhythmogenesis. Therefore, FGF23-induced I_Na-Late_ may contribute to FGF23-related PV arrhythmogenesis. Ranolazine has been shown to prolong ventricular APD and QTc interval by the inhibitory effect on rapidly activating delayed rectifier potassium current blockers. [32, 33] In this study, as the tracing shown in Figure 3B, ranolazine shortened the APD in control and FGF23-treated PV cardiomyocytes. Those findings are similar to the results in the previous studies of ranolazine on ventricular middle myocardium, Purkinje fibers, and PV cardiomyocytes. [29, 32] The ranolazine-induced APD shortening may be caused by the decrease in net inward current due to I_Na-Late_ inhibition.

Similar to those found in mice atrial cell lines (HL-1 cells), we found that FGF23 increased the L-type calcium current and calcium transients in PV cardiomyocytes. [10, 11] FGF23-increased calcium transients in PV cardiomyocytes may be induced by enhanced I_Ca-L_, NCX, and I_Na-Late_ currents. [34, 35] Moreover, FGF23-treated PV cardiomyocytes had increased phosphorylation of CaMKII, which can hyperphosphorylate the RyR resulting in increased calcium leakage. [13, 36] We found that FGF23 induced mitochondrial ROS genesis in PV cardiomyocytes. These results indicated that FGF23 can contribute to the ROS overproduction in CKD and result in calcium dysregulation *via* increased phosphorylation of CaMKII. [18, 25, 37, 38] The increase of I_Na-Late_ was demonstrated to enhance mitochondrial oxidative stress. [25] We found that ranolazine could block the effects of FGF23 on mitochondrial ROS. Accordingly, as speculated in Figure 7C, FGF23 may produce ROS resulting in calcium dysregulation through activation of the I_Na-Late_. Our findings imply the therapeutic potential of ranolazine on CKD-related arrhythmogenesis.

Phospholipase Cγ-PKC and phospholipase Cγ-calcineurin pathways has been shown to mediate FGF23-induced ventricular hypertrophy. [38] However, this study found similar expressions of calcineurin in control and FGF23-treated PV cardiomyocytes. Therefore, phospholipase Cγ-calcineurin pathway is not involved in FGF23-triggered PV arrhythmogenesis. Increased activity of PKC or CaMKII was proven to enhance the I_Na-Late_ leading to calcium overload. [20, 22, 39] In this study, an inhibitor of PKC only reduced the I_Na-Late_ in FGF23-treated cells which suggests that FGF23 may increase the I_Na-Late_ by PKC (Figure 7C). However, a selective CaMKII blocker reduced the I_Na-Late_ in control and FGF23-treated cells. Therefore, PKC and CaMKII inhibitors both potentially can reduce AF risk in CKD patients.

This study has some limitations. We studied the acute effects of FGF23 on PV electrical activity, but the long-term effects of FGF23 on the cardiovascular system may be different. Although this study tried to dissect the molecular mechanism underlying FGF23-induced PV arrhythmogenesis, interactions of ROS, I_Na-Late_, PKC, and CaMKII were not completely elucidated. Moreover, we studied the effects of FGF23 on PV cardiomyocytes in healthy animals. However, it is not clear whether our findings can be translated to pathological settings.

In conclusion, FGF23 induces PV arrhythmogenesis with enhanced NCX and calcium transient through increasing the I_Na-Late_, oxidative stress, and PKC activation.

## MATERIALS AND METHODS

### Isolation of single cardiomyocytes and cell preparations

The investigation was approved by the local ethics review board and conformed to the institutional Guide for the Care and Use of Laboratory Animals published by the US National Institutes of Health. Male rabbits (2.0~3.0 kg) were anesthetized with an intraperitoneal injection of sodium pentobarbital (100 mg/kg) and heparin (2500 units). Single cardiomyocytes from rabbit PVs were enzymatically dissociated through a previously described procedure. [40] In brief, a mid-line thoracotomy was performed, and the heart and lungs were removed. PVs were perfused in a retrograde manner *via* polyethylene tubing cannulated through the aorta and left ventricle into the left atrium. The free end of the polyethylene tube was connected to a Langendroff perfusion column for perfusion with oxygenated normal Tyrode's solution (containing (in mM): NaCl 137, KCl 5.4, CaCl_2_ 1.8, MgCl_2_ 0.5, HEPES 10 and glucose 11; with pH adjusted to 7.4 by titrating with 1 N NaOH). The perfusate was replaced with oxygenated Ca^2+^-free Tyrode's solution containing 300 units/ml collagenase (Sigma Type I) and 0.25 units/ml protease (Sigma, Type XIV) for 8~12 min. Proximal PVs were cut away from the atrium and lung, and were gently shaken in 5~10 ml of Ca^2+^-free oxygenated Tyrode's solution until single cardiomyocytes were obtained. The solution was then gradually changed to oxygenated normal Tyrode's solution. Cells were allowed to stabilize in the bath for at least 30 min before the experiments. Single cardiomyocytes with spontaneous activity were identified by the presence of constant beating during perfusion with Tyrode's solution. PV cardiomyocytes were pretreated in the control and FGF23 (0.1 and 1 ng/mL) incubated for 4~6 h with or without ranolazine (10 μM), chelerythrine chloride (2.5 μM; sigma, St. Louis, MO, USA), KN93 (1 μM; Calbiochem, San Diego, CA, USA), and KN92 (1 μM; Calbiochem) before being harvested for further analysis.

### Patch clamp experiments

A whole-cell patch-clamp was used on isolated PV cardiomyocytes with an Axopatch 1D amplifier (Axon Instruments, Foster City, CA, USA) at 35±1C. [40] The ionic currents and action potentials (APs) were recorded at an approximately similar period (3~5 min) after rupture or perforation to avoid decay of ion channel activity over time. The micropipette resistance was 3~5 MΩ. A small hyperpolarizing step from a holding potential of −50 mV to a testing potential of −55 mV for 80 ms was delivered at the beginning of each experiment. The area under the capacitative current curve was divided by the applied voltage step to obtain the total cell capacitance. Normally, 60%~80% series resistance (R_s_) was electronically compensated for. APs were recorded in the current-clamp mode and ionic currents in the voltage-clamp mode. Spontaneous beating rate was defined as the constant occurrence of spontaneous APs in the absence of any electrical stimuli. DADs were defined as the presence of spontaneous depolarization of the impulse after full repolarization had occurred. The DADs were selected from consistent deflections without abrupt changes of the resting membrane potential and AP morphology.

Micropipettes were filled with a solution containing (in mM) CsCl 130, MgCl_2_ 1, MgATP 5, HEPES 10, NaGTP 0.1, and Na2 phosphocreatine 5, titrated to a pH of 7.2 with CsOH for the experiments on the L-type calcium current (I_Ca-L_); with a solution containing (in mM) KCl 20, K aspartate 110, MgCl_2_ 1, MgATP 5, HEPES 10, EGTA 0.5, NaGTP 0.1, and Na_2_ phosphocreatine 5, titrated to a pH of 7.2 with KOH for experiments on the AP; with a solution containing (in mM) NaCl 20, CsCl 110, MgCl_2_ 0.4, CaCl_2_ 1.75, tetraethylammonium chloride (TEACl) 20, BAPTA 5, glucose 5, MgATP 5, and HEPES 10, titrated to a pH of 7.25 with CsOH for experiments on the NCX current; and with a solution containing (in mM) CsCl 130, Na_2_ATP 4, MgCl_2_ 1, EGTA 10, and HEPES 5 at a pH of 7.3 with NaOH for the I_Na-Late_.

The I_Na-Late_ was recorded at room temperature with an external solution containing (in mM): NaCl 130, CsCl 5, MgCl_2_ 1, CaCl_2_ 1, HEPES 10, and glucose 10 at a pH of 7.4 with NaOH by a step/ramp protocol (−100 mV stepped to +20 mV for 100 ms, then ramped back to −100 mV over 100 ms). The I_Na-late_ was measured as the tetrodotoxin (30 μM)-sensitive portion of the current traces obtained when the voltage was ramped back to −100 mV.

The I_Ca-L_ was measured as an inward current during depolarization from a holding potential of −50 mV to test potentials ranging from −40 to +60 mV in 10-mV steps for 300 ms at a frequency of 0.1 Hz using a perforated patch clamp with amphotericin B. NaCl and KCl in the external solution were respectively replaced with tetraethylammonium chloride and CsCl. In order to avoid ‘run-down’ effects, the I_Ca-L_ was measured at 5~15 min after rupturing the membrane patch in each PV cardiomyocyte.

The NCX current was elicited by test pulses of between −100 and +100 mV from a holding potential of −40 mV for 300 ms at a frequency of 0.1 Hz. The amplitudes of the NCX current were measured as 10-mM nickel-sensitive currents. The external solution (in mM) consisted of NaCl 140, CaCl_2_ 2, MgCl_2_ 1, HEPES 5, and glucose 10 with a pH of 7.4, and contained strophanthidin (10 μM), nitrendipine (10 μM), and niflumic acid (100 μM).

### Measurement of intracellular and SR calcium contents

As described previously, PV cardiomyocytes were loaded with fluorescent Ca^2+^ (10 μM, fluo-3/AM) for 30 min at room temperature. [41, 42] The Fluo-3 fluorescence was excited with a 488-nm line of an argon ion laser. The emission was recorded at > 515 nm. Cells were repetitively scanned at 2-ms intervals. Fluorescence imaging was performed with a laser scanning confocal microscope (Zeiss LSM 510, Carl Zeiss, Jena, Germany) and an inverted microscope (Axiovert 100, Carl Zeiss). The fluorescent signals were corrected for variations in dye concentrations by normalizing the fluorescence (F) against the baseline fluorescence (F_0_), to obtain reliable information about transient intracellular Ca2+ (Ca2+i) changes from baseline values ((F - F_0_)/F_0_) and to exclude variations in the fluorescence intensity by different volumes of injected dye. The Ca^2+^i transient, peak systolic Ca^2+^i, and diastolic Ca^2+^i were measured during a 2-Hz field-stimulation with 10-ms twice-threshold strength square-wave pulses.

After achieving steady-state Ca^2+^ transients with repeated pulses from −40 to 0 mV (1 Hz for 5 s), the total amount of charge crossing the membrane SR Ca^2+^ content (Ccaff) was estimated by integrating the NCX current rapid application of 20 mM caffeine during rest with the membrane potential clamped to −40 mV to cause SR Ca^2+^ release. The NCX current was measured by whole-cell patch-clamp experiments. The total SR Ca^2+^ content (expressed as mM of cytosol) was determined using of the equation: SR Ca^2+^ content = [(1 + 0.12) (Ccaff/F × 1000)]/(Cm × 8.31 × 6.44), where Cm = membrane capacitance, F = Faraday's number, and the cell surface to volume ratio = 6.44 pF/pL.

### Western blot analysis

Control and FGF23 (1 ng/mL)-treated PV cardiomyocytes were centrifuged and washed with cold PBS, and lysed on ice for 30 min in RIPA buffer containing 50 mM Tris, pH 7.4, 150 mM NaCl, 1% NP40, 0.5% sodium deoxycholate, 0.1% sodium dodecylsulfate (SDS), and protease inhibitor cocktails (Sigma-Aldrich, St. Louis, MO, USA). The protein concentration were determined with a Bio-Rad protein assay reagent (Bio-Rad, Hercules, CA, USA). Proteins were separated in 4%~12% SDS- polyacrylamide gel electrophoresis (PAGE) under reducing conditions and electrophoretically transferred into an equilibrated polyvinylidene difluoride membrane (Amersham Biosciences, Buckinghamshire, UK). All blots were probed with primary antibodies against SERCA2a (Santa Cruz Biotechnology, Santa Cruz, CA, USA), pCaMKII at Thr 286, calcineurin (Abcam, Cambridge, United Kingdom), CaMKII, PLB pSer16 (GeneTex, San Antonio, TX, USA), RyR pS2808, RyR pS2814, PLB pThr17 (Badrilla, Leeds, UK), RyR channels, total PLB (Thermo, Rockford, IL, USA), α-actin (Sigma, St. Louis, MO, USA), and all secondary antibodies conjugated with horseradish peroxidase. All bound antibodies were detected with an enhanced chemiluminescence detection system (Millipore, Billerica, MA, USA) and analyzed with AlphaEaseFC software. All targeted bands were normalized to α-actin to confirm equal protein loading.

### Measurement of intracellular ROS

We used the mitochondria-specific ROS indicator MitoSOX Red to assess ROS production in mitochondria of control and FGF23-treated PV cardiomyocytes. Experiments were also performed using a laser scanning confocal microscope (Zeiss LSM 510, Carl Zeiss) and an inverted microscope (Axiovert 100) with a 60x1.4 numerical aperture oil immersion objective as described previously. [25] Cardiomyocytes were maintained in oxygenated normal Tyrode's solution (containing (in mM): NaCl 137, KCl 5.4, CaCl_2_ 1.8, MgCl_2_ 0.5, HEPES 10 and glucose 11; with the pH adjusted to 7.4 by titrating with 1 N NaOH) supplemented with the appropriate fluorescent dye of 5 μM MitoSOX Red (life Technologies, Grand Island, NY, USA). MitoSOX Red was excited at 488 nm and fluorescence signals were acquired at wavelengths of > 505 nm in the XY mode of the confocal system. Fluorescent images were analyzed using Image-Pro plus 6.0 and Sigmaplot 12.3 software.

### Statistical analysis

All continuous parameters are expressed as the mean ± standard error of the mean (SEM). A one-way or two-way repeated analysis of variance (ANOVA) with a Bonferroni post-hoc test or an unpaired t-test was used to compare differences in control and FGF23-treated PV cardiomyocytes before and after ranolazine, KN93, KN92, or chelerythrine chloride when appropriate. A p value of < 0.05 was considered statistically significant.

## References

[1] Krahn AD, Manfreda J, Tate RB, Mathewson FA, Cuddy TE (1995). The natural history of atrial fibrillation: incidence, risk factors, and prognosis in the Manitoba Follow-Up Study. Am J Med.

[2] Kannel WB, Abbott RD, Savage DD, McNamara PM (1982). Epidemiologic features of chronic atrial fibrillation: the Framingham study. N Engl J Med.

[3] Chen YC, Chen SA, Chen YJ, Chang MS, Chan P, Lin CI (2002). Effects of thyroid hormone on the arrhythmogenic activity of pulmonary vein cardiomyocytes. J Am Coll Cardiol.

[4] Heine GH, Seiler S, Fliser D (2012). FGF-23: the rise of a novel cardiovascular risk marker in CKD. Nephrol Dial Transplant.

[5] Mathew JS, Sachs MC, Katz R, Patton KK, Heckbert SR, Hoofnagle AN, Alonso A, Chonchol M, Deo R, Ix JH, Siscovick DS, Kestenbaum B, de Boer IH (2014). Fibroblast growth factor-23 and incident atrial fibrillation: the Multi-Ethnic Study of Atherosclerosis (MESA) and the Cardiovascular Health Study (CHS). Circulation.

[6] Ananthapanyasut W, Napan S, Rudolph EH, Harindhanavudhi T, Ayash H, Guglielmi KE, Lerma EV (2010). Prevalence of atrial fibrillation and its predictors in nondialysis patients with chronic kidney disease. Clin J Am Soc Nephrol.

[7] Soliman EZ, Prineas RJ, Go AS, Xie D, Lash JP, Rahman M, Ojo A, Teal VL, Jensvold NG, Robinson NL, Dries DL, Bazzano L, Mohler ER (2010). Chronic kidney disease and prevalent atrial fibrillation: the Chronic Renal Insufficiency Cohort (CRIC). Am Heart J.

[8] Fukunaga N, Takahashi N, Hagiwara S, Kume O, Fukui A, Teshima Y, Shinohara T, Nawata T, Hara M, Noguchi T, Saikawa T (2012). Establishment of a model of atrial fibrillation associated with chronic kidney disease in rats and the role of oxidative stress. Heart Rhythm.

[9] Gutierrez OM, Mannstadt M, Isakova T, Rauh-Hain JA, Tamez H, Shah A, Smith K, Lee H, Thadhani R, Juppner H, Wolf M (2008). Fibroblast growth factor 23 and mortality among patients undergoing hemodialysis. N Engl J Med.

[10] Kao YH, Chen YC, Lin YK, Shiu RJ, Chao TF, Chen SA, Chen YJ (2014). FGF-23 dysregulates calcium homeostasis and electrophysiological properties in HL-1 atrial cells. Eur J Clin Invest.

[11] Touchberry CD, Green TM, Tchikrizov V, Mannix JE, Mao TF, Carney BW, Girgis M, Vincent RJ, Wetmore LA, Dawn B, Bonewald LF, Stubbs JR, Wacker MJ (2013). FGF23 is a novel regulator of intracellular calcium and cardiac contractility in addition to cardiac hypertrophy. Am J Physiol Endocrinol Metab.

[12] Korantzopoulos PG, Goudevenos JA (2009). Atrial fibrillation in end-stage renal disease: an emerging problem. Kidney Int.

[13] Chen WT, Chen YC, Hsieh MH, Huang SY, Kao YH, Chen YA, Lin YK, Chen SA, Chen YJ (2015). The uremic toxin indoxyl sulfate increases pulmonary vein and atrial arrhythmogenesis. J Cardiovasc Electrophysiol.

[14] Chen SA, Hsieh MH, Tai CT, Tsai CF, Prakash VS, Yu WC, Hsu TL, Ding YA, Chang MS (1999). Initiation of atrial fibrillation by ectopic beats originating from the pulmonary veins: electrophysiological characteristics, pharmacological responses, and effects of radiofrequency ablation. Circulation.

[15] Chen YJ, Chen SA, Chen YC, Yeh HI, Chan P, Chang MS, Lin CI (2001). Effects of rapid atrial pacing on the arrhythmogenic activity of single cardiomyocytes from pulmonary veins: implication in initiation of atrial fibrillation. Circulation.

[16] Chen YC, Pan NH, Cheng CC, Higa S, Chen YJ, Chen SA (2009). Heterogeneous expression of potassium currents and pacemaker currents potentially regulates arrhythmogenesis of pulmonary vein cardiomyocytes. J Cardiovasc Electrophysiol.

[17] Huang SY, Chen YC, Kao YH, Hsieh MH, Chen YA, Chen WP, Lin YK, Chen SA, Chen YJ (2015). Renal failure induces atrial arrhythmogenesis from discrepant electrophysiological remodeling and calcium regulation in pulmonary veins, sinoatrial node, and atria. Int J Cardiol.

[18] Scialla JJ, Wolf M (2014). Roles of phosphate and fibroblast growth factor 23 in cardiovascular disease. Nat Rev Nephrol.

[19] Wagner S, Rokita AG, Anderson ME, Maier LS (2013). Redox regulation of sodium and calcium handling. Antioxid Redox Signal.

[20] Makielski JC (2016). Late sodium current: A mechanism for angina, heart failure, and arrhythmia. Trends Cardiovasc Med.

[21] De Windt LJ, Lim HW, Haq S, Force T, Molkentin JD (2000). Calcineurin promotes protein kinase C and c-Jun NH2-terminal kinase activation in the heart. Cross-talk between cardiac hypertrophic signaling pathways. J Biol Chem.

[22] Wu Y, Wang L, Ma J, Song Y, Zhang P, Luo A, Fu C, Cao Z, Wang X, Shryock JC, Belardinelli L (2015). Protein kinase C and Ca(2+) -calmodulin-dependent protein kinase II mediate the enlarged reverse INCX induced by ouabain-increased late sodium current in rabbit ventricular myocytes. Exp Physiol.

[23] Six I, Okazaki H, Gross P, Cagnard J, Boudot C, Maizel J, Drueke TB, Massy ZA (2014). Direct, acute effects of Klotho and FGF23 on vascular smooth muscle and endothelium. PLoS One.

[24] Conn PM (2006). Handbook of models for human aging.

[25] Viatchenko-Karpinski S, Kornyeyev D, El-Bizri N, Budas G, Fan P, Jiang Z, Yang J, Anderson ME, Shryock JC, Chang CP, Belardinelli L, Yao L (2014). Intracellular Na+ overload causes oxidation of CaMKII and leads to Ca2+ mishandling in isolated ventricular myocytes. J Mol Cell Cardiol.

[26] Gao Z, Chen B, Joiner ML, Wu Y, Guan X, Koval OM, Chaudhary AK, Cunha SR, Mohler PJ, Martins JB, Song LS, Anderson ME (2010). I(f) and SR Ca(2+) release both contribute to pacemaker activity in canine sinoatrial node cells. J Mol Cell Cardiol.

[27] Antzelevitch C, Burashnikov A (2011). Overview of Basic Mechanisms of Cardiac Arrhythmia. Card Electrophysiol Clin.

[28] Greenspan K, Edmands RE, Fisch C (1967). Effects of cycle-length alteration on canine cardiac action potentials. Am J Physiol.

[29] Sicouri S, Glass A, Belardinelli L, Antzelevitch C (2008). Antiarrhythmic effects of ranolazine in canine pulmonary vein sleeve preparations. Heart Rhythm.

[30] Chang SL, Chen YC, Yeh YH, Lin YK, Wu TJ, Lin CI, Chen SA, Chen YJ (2011). Heart failure enhanced pulmonary vein arrhythmogenesis and dysregulated sodium and calcium homeostasis with increased calcium sparks. J Cardiovasc Electrophysiol.

[31] Belardinelli L, Shryock JC, Fraser H (2006). Inhibition of the late sodium current as a potential cardioprotective principle: effects of the late sodium current inhibitor ranolazine. Heart.

[32] Antzelevitch C, Belardinelli L, Zygmunt AC, Burashnikov A, Di Diego JM, Fish JM, Cordeiro JM, Thomas G (2004). Electrophysiological effects of ranolazine, a novel antianginal agent with antiarrhythmic properties. Circulation.

[33] Chaitman BR, Pepine CJ, Parker JO, Skopal J, Chumakova G, Kuch J, Wang W, Skettino SL, Wolff AA, Combination Assessment of Ranolazine In Stable Angina I (2004). Effects of ranolazine with atenolol, amlodipine, or diltiazem on exercise tolerance and angina frequency in patients with severe chronic angina: a randomized controlled trial. JAMA.

[34] Fabiato A (1983). Calcium-induced release of calcium from the cardiac sarcoplasmic reticulum. Am J Physiol.

[35] Venetucci LA, Trafford AW, O'Neill SC, Eisner DA (2007). Na/Ca exchange: regulator of intracellular calcium and source of arrhythmias in the heart. Ann N Y Acad Sci.

[36] Tsai WC, Yang LY, Chen YC, Kao YH, Lin YK, Chen SA, Cheng CF, Chen YJ (2013). Ablation of the androgen receptor gene modulates atrial electrophysiology and arrhythmogenesis with calcium protein dysregulation. Endocrinology.

[37] Schepers E, Glorieux G, Dhondt A, Leybaert L, Vanholder R (2009). Role of symmetric dimethylarginine in vascular damage by increasing ROS via store-operated calcium influx in monocytes. Nephrol Dial Transplant.

[38] Faul C, Amaral AP, Oskouei B, Hu MC, Sloan A, Isakova T, Gutierrez OM, Aguillon-Prada R, Lincoln J, Hare JM, Mundel P, Morales A, Scialla J (2011). FGF23 induces left ventricular hypertrophy. J Clin Invest.

[39] Makielski JC, Farley AL (2006). Na(+) current in human ventricle: implications for sodium loading and homeostasis. J Cardiovasc Electrophysiol.

[40] Chen YC, Kao YH, Huang CF, Cheng CC, Chen YJ, Chen SA (2010). Heat stress responses modulate calcium regulations and electrophysiological characteristics in atrial myocytes. J Mol Cell Cardiol.

[41] Chang SH, Chen YC, Chiang SJ, Higa S, Cheng CC, Chen YJ, Chen SA (2008). Increased Ca(2+) sparks and sarcoplasmic reticulum Ca(2+) stores potentially determine the spontaneous activity of pulmonary vein cardiomyocytes. Life Sci.

[42] Lkhagva B, Chang SL, Chen YC, Kao YH, Lin YK, Chiu CT, Chen SA, Chen YJ (2014). Histone deacetylase inhibition reduces pulmonary vein arrhythmogenesis through calcium regulation. Int J Cardiol.

